# Weighted Global Artificial Bee Colony Algorithm Makes Gas Sensor Deployment Efficient

**DOI:** 10.3390/s16060888

**Published:** 2016-06-16

**Authors:** Ye Jiang, Ziqing He, Yanhai Li, Zhengyi Xu, Jianming Wei

**Affiliations:** 1 Shanghai Advanced Research Institute, Chinese Academy of Sciences, Shanghai 201210, China; jiangye@sari.ac.cn (Y.J.); hezq@sari.ac.cn (Z.H.); liyh@sari.ac.cn (Y.L.); xuzy@sari.ac.cn (Z.X.); 2 University of Chinese Academy of Sciences, Beijing 100049, China

**Keywords:** artificial bee colony algorithm, global factor, convergence speed, benchmark function, gas diffusion model, optimal deployment

## Abstract

This paper proposes an improved artificial bee colony algorithm named Weighted Global ABC (WGABC) algorithm, which is designed to improve the convergence speed in the search stage of solution search equation. The new method not only considers the effect of global factors on the convergence speed in the search phase, but also provides the expression of global factor weights. Experiment on benchmark functions proved that the algorithm can improve the convergence speed greatly. We arrive at the gas diffusion concentration based on the theory of CFD and then simulate the gas diffusion model with the influence of buildings based on the algorithm. Simulation verified the effectiveness of the WGABC algorithm in improving the convergence speed in optimal deployment scheme of gas sensors. Finally, it is verified that the optimal deployment method based on WGABC algorithm can improve the monitoring efficiency of sensors greatly as compared with the conventional deployment methods.

## 1. Background

Swarm intelligence appeared in the 1980s as a new research project, and has aroused the attention of the researchers in many fields. The study of swarm intelligence mainly originated from the behavior research on social insects such as ants [1] and bees [2]. The researchers extract models, establish rules and finally obtain related algorithms from the group activities of the insects. The obtained algorithms can be used to solve complex problems by a large number of simple individuals and show the advantages of flexibility, efficiency, and robustness. Meanwhile, a global model is not necessary for the algorithms. Representative algorithms include ant colony optimization (ACO) algorithm, genetic algorithm (GA), particle swarm optimization (PSO) algorithm, and artificial bee colony (ABC) algorithm, *etc*.

Artificial bee colony (ABC) algorithm first proposed by Karaboga in 2005 is a new intelligent swarm optimization algorithm [2], which has shown more advantages as compared with some conventional biological-inspired algorithms, such as genetic algorithm (GA) [3], and ant colony algorithm [4]. In view of such advantages of the ABC algorithm as less control parameters, simpler principle and stronger robustness, it has been applied to function optimization, artificial network training, production scheduling, path planning and so on. The improved ABC algorithm is applied to the gravity matching navigation field by Gao *et al.* [5] so as to meet the need for high accuracy in gravity aided navigation. The artificial bee colony (ABC) optimization approach is applied to the data gathered from these sensors to help instructors understand their students’ reading concentration rates in a classroom environment [6], the results of which show that the use of the ABC algorithm in the proposed system can effectively obtain near-optimal solutions. The ABC algorithm is used to optimize the network configuration distribution to make network loss minimal by Rao *et al.* [7], the ABC algorithm [8,9] is applied to the neural network training, the ABC algorithm is used to solve the constraint optimization problems [10], and also many optimization and application problems [11,12,13].

However, the ABC algorithm has the defects of slow convergence speed in the search stage, and ease to fall into local optimization. To this end, some scholars put forward many improved methods for ABC algorithm. Akay and Karaboga [14] added two new control parameters MR for speeding up the convergence speed and SF for controlling the noise influence on the algorithm. Zhu and Kwong [15] proposed an improved ABC algorithm, called gbest-guided ABC (GABC) algorithm, by incorporating the information of global best (gbest) solution into the ABC algorithm to improve the exploitation. Shah *et al.* [16] applied the GABC algorithm to Neural Network (NN) training. Garg *et al.* [17] applied the GABC algorithm to the load flow problem for five bus networks. The GABC algorithm is applied to optimize the emission and overall cost of operation of wind–thermal power system [18]. Zhang *et al.* [19] put forward three kinds of improved ABC algorithms, which can obtain more accurate optimization results, and Alatas [20] used the theory of chaos map to choose the parameters of search equation, which can prevent the algorithm from falling into local optimization. Articles [21,22,23,24] introduced some strategies based on ABC algorithm to solve the structure problems. Articles [25,26,27,28,29,30,31,32,33] also improved the ABC algorithm to different extents, and the improved algorithms demonstrated their effectiveness using benchmark functions [34,35,36,37].

However, there is still some insufficiency in the ABC algorithm regarding its solution search equation, which is good at exploration but poor at exploitation. Inspired by PSO and based on the article [15], we propose an improved ABC algorithm considering the global factor to the exploitation equation called Weighted-Global ABC, which not only takes into account the impact of global factors on the rate of convergence, but also adds a weight factor, such that at the beginning of the iteration, the weight value is large and the convergence speed is fast, while at the end of the iteration, the weight value decreases and the algorithm has a good local convergence ability.

Deploying a wireless sensor network, we should bear in mind the major factors which are gas characteristics, environmental characteristics, and features of communication. During the process, we preferred considering communication factors generally. At present, there are a lot of international studies in this aspect, which are mainly related to prolonging the network lifetime, increasing the coverage range, reducing the number of deployment sensors, improving the detection efficiency, reducing energy consumption, and *etc.* Articles [38,39,40,41,42,43,44] are related to methods simply based on the communication characteristics. Although the method can perceive information from the sensing unit effectively and then get it back to the monitoring center, it did not take the actual environmental factors into consideration. As a result, it is difficult to ensure accurate perception to the environment in a real environment. These ideas may not be practical due to the influence of meteorological factors and topographic factors.

Aimed at solving the problem of hazard gas diffusion in chemical industrial park, there are also many combination gas diffusion models [45] used to study the deployment of gas sensors, mainly the heuristic analysis method [46] at present, which is only for the serious leakage scenario, without considering the uncertainty of the leakage scenario. Besides, there are some methods based on the theory of risk, this method is proposed by the international standard committee, risk and its minimization is the objective function, the disadvantage is that the method can only get local optimal solution. Mixed integer linear programming, by Legg and others, according to the sensor deployment scheme of water pollution considered the impact of node failure on the network, but it is based on the assumption of node failure probability and does not take into account the time series, and is not in conformity with the actual situation. The methods above are based on the simple diffusion model with no consideration given to environmental features such as wind speed and direction, so the node deployment is not accurate.

Miyata and Mori [47] mainly considered the influence of wind direction on the deployment plan, and the sensor node deployment target in that article is that the number of alarm nodes is not less than two in different wind directions under the condition of gas leak. The deployment process is to, firstly, locate the leakage source and initial deployment and determine a threshold leakage rate, then determine the shortest distance between the security region and the leakage source (which is the same to wind direction), and, thirdly, to determine the threshold leakage rate according to the gas diffusion model. This model is simulated using CFD simulate tools with different leakage rate combining ERPG-2 threshold level [48], the leakage rate reaches the threshold concentration level in the shortest distance, namely threshold leak rate. The leakage rate that is less than the threshold rate will not produce harm. When the leakage rate is greater than the threshold rate, there exist dangers. At last, we use the knowledge to evaluate the right node location. The article [49] is a plan for an offshore oil storage facility in the leak case. The deployment target is to achieve a maximum efficiency with the limited sensors. As to environmental factors, we mainly consider wind direction, wind speed, leakage rate, and leakage location. It simulates the gas diffusion using CFD tools, then samples 50 leakage scenarios, meshes the area, and finally deploys a node in the center of each grid. If the leakage happens, each sensor is sorted in descending order according to the detected gas concentration, then with limited sensors, the topmost positions are selected as the optimal deployment positions. Since the method above—based on the CFD diffusion model—takes many factors into account, it is possible to produce a diffusion model closer to the actual situation. However, it needs a long time to run, the hardware requirements are also quite high.

So, combining environmental properties with gas properties to optimize the deployment plan is the most practical, highest efficient and most accurate way. To solve the problem above, this paper puts forward a gas sensor optimal deployment method based on the improved artificial bee colony (ABC) algorithm, which not only combines the gas diffusion model, but also considers the influence of buildings into the diffusion model. We can get the deployment scheme through actual application. So the method is of a certain practical significance, and at the same time can provide a reference to the current deployment method.

The next section of this paper is to first introduce the artificial colony algorithm, and then propose an improved artificial colony algorithm which is called Weighted Global ABC algorithm, the gas diffusion model combined with the influence of buildings, and a new type of gas sensor deployment optimization method using the improved artificial colony algorithm. At last, it is verified through experiments that the scheme can improve the monitoring efficiency significantly as compared with the deployment schemes according to the standards.

## 2. The Basic Artificial Bee Colony Algorithm

There are three kinds of bees in the artificial bee colony algorithm, namely employed bees, onlookers, and scouts, wherein the employed bees go to the food source for honey, the food source is equal to the feasible solution of the objective function, and the number of food sources is equal to the number of feasible solutions, meanwhile, the number of food sources is equal to the number employed bees. Then the onlooker bees choose a food source randomly to compare the probability in the whole foods for crossover and mutation, the function of scouts is to observe whether the food source can be optimized within the limited times, or otherwise the food source would be abandoned, and then the scout bees become the employed bees with a new food source produced.

The process is described as follows:

(1) Employed bee phase

There are two stages in the employed bee stage, the first stage is initialization, in which every food source serves as a feasible solution, and the parameters of the feasible solution are initialized randomly, different function solutions are obtained by computing the objective function with the feasible solution, and then the corresponding fitness is obtained accordingly by putting the function solution into the fitness function, described by Equation (1) as follows:

(1) $$fitness\left\{ \begin{array}{l} =1/(1+fi)\\ =1+abs(fi) \end{array}\right.\;\begin{array}{l} fi\geq 0\\ fi<0 \end{array}$$

where fi means the value of the objective function.

The second stage is crossover and mutation, making the crossover and mutation to the jth element of every food source with the jth element of its neighbor food source, described by Equation (2) as follows:

(2) $$V_{ij}=x_{ij}+\phi_{ij}(x_{i}{}_{j}-x_{k}{}_{j})$$

where $V_{ij}$ denotes the solution after crossover and mutation, $x_{ij}$ denotes the jth element of the ith, $x_{kj}$ denotes the jth element of the *k*th solution, and k is neighbor solution of ith solution and it is different from i, $\phi_{ij}$ is a random number within the range of [−1, 1]. Then calculate the fitness after crossover and mutation using Equation (1), and compare the calculated fitness with the initial fitness, if the latter is better, then change the parameters to be the latter parameters.

(2) Onlookers phase

Get the mutated fitness, then calculate the probability according to the Equation (3):

(3) $$P_{i}=\frac{fitness_{i}}{\sum_{i=1}^{SN}fitness_{i}}$$

$fitness_{i}$ denotes the fitness of the every solution, SN denotes the number of the food sources, we can get the probability of every solution according to Equation (3). Then generate a number in the range of [0~1] randomly, compare the number with the probability, if the number is less than the probability, then continue to cross and mutate.

(3) Scout bee phase

If the feasible solution cannot converge within limited times of crossover and mutation, then abandon the feasible solution, and iterate with a new solution randomly.

## 3. Weighted Global ABC (WGABC) Algorithm

The article [15] proposed a GABC algorithm considering the global factor and taking advantage of the information of the global best (gbest) solution to guide the search of candidate solutions, described by Equation (4) as follows:

(4) $$V_{ij}=x_{ij}+\phi_{ij}(x_{i}{}_{j}-x_{k}{}_{j})+\psi_{ij}(y_{j}-x_{i}{}_{j})$$

where $y_{j}$ denotes the jth element of the global optimum solution, $\psi_{ij}$ is a random number in the range of [0,2], but the algorithm cannot improve the convergence speed greatly.

In order to improve the exploitation more efficiently, we continue to propose a new algorithm called Global ABC based on the GABC, described by Equation (5) as follows:

(5) $$V_{ij}=(0.9-iter\times 0.5/max\;cycle)\times y_{j}+\varphi_{ij}(x_{ij}-x_{kj})+(0.9-iter\times 0.5/max\;cycle)\times (y_{j}-x_{ij})$$

where iter is the iterth iteration, maxcycle is the maximum iteration.

0.9−iter×0.5/maxcylce is the weighting factor, $y_{j}$ is the jth element of the global optimum solution, Equation (5) considers the global factor into the ABC algorithm.

At the beginning of the iteration, the weighting factor is larger, and the global convergence speed is faster. Since the smaller weights ensure good convergence rate for local search, weight coefficient should become smaller and smaller as the number of iterations increases.

**Figure 1:**
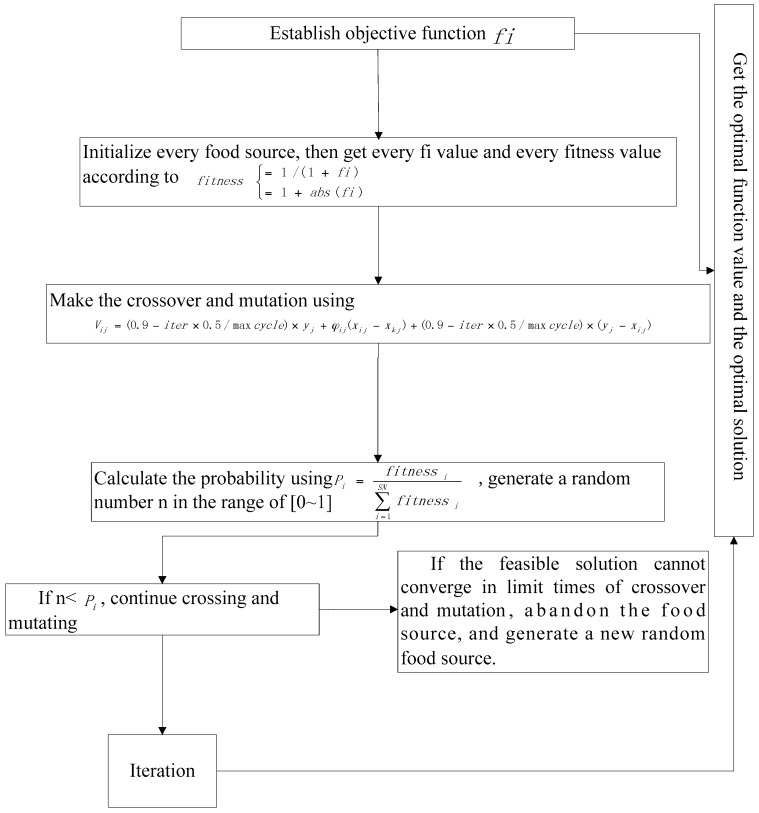
The flowchart of the WGABC algorithm.

The flowchart is given in Figure 1.

In Equation (5), the first term and the third term in the right-hand side of Equation (5) are the new added terms, called gbest terms, y_j_ is the jth element of the global best solution, 0.9−iter×0.5/maxcylce is the weighting factor.

Firstly, the gbest terms can expand the search scope of the solution, then reduce the probability of falling into local optimization, therefore, they can improve the convergence precision. Secondly, the gbest terms can drive the new candidate solution towards the global best solution, therefore, they can improve the convergence speed. Thirdly, the weighting factor is very significant. At the beginning of the iteration, the weighting factor is large, which has good convergence speed for global search, with the increase of the number of iterations, the weighting factor decreases, which has good convergence speed for local search.

Therefore, Equation (1) can improve the exploitation ability, convergence precision, and convergence speed of ABC algorithm perfectly.

Although the WPSO algorithm can enhance the global search ability and the local search ability, the effect of the WGABC algorithm is better than WPSO algorithm in the convergence speed and the convergence precision.

## 4. Experiments of Algorithm Validation and Analysis

### 4.1. Benchmark Functions

In order to test the convergence speed of the improved algorithm in this thesis, we particularly select six mainstream benchmark functions for test, which are shown in Table 1.

Wherein D denotes the dimension of the solution space of each function, and indicates different values due to the benchmark functions [34,35,36,37,38,39,40,41,42,43,44,45,46,47]. The value of D can be 2 or 3 in Rosenbrock function, and the value of D may be 30 or 60 in other functions. Akay and Karaboga [50] point out that it is not necessary to have a large number of the food sources in the artificial colony algorithm, therefore the number of food sources in this experiment is 20. According to the article [34], the upper limit of the times of crossover and mutation of each food source is limit = D × SN, wherein SN denotes the number of food sources, namely the number of feasible solutions, D denotes the dimension of every feasible solution and the limit can prevent crossover and mutation from falling into an infinite loop. When a food source is not convergent after limited times of crossover and mutation, this food source can be abandoned, and then a random solution is generated to continue the crossover and mutation. In order to compare the convergence precision of different algorithms, here, we compare WGABC algorithm with ABC algorithm, GABC algorithm, PSO algorithm, and DE algorithm. The number of food sources is 20 in all ABC algorithms, meanwhile, the number of particles is 20 in PSO algorithm, and the number of populations in DE algorithm is 20. The number of iterations is 2500 in each experiment, and each experiment is repeated 30 times using the MATLAB software simulation.

### 4.2. Experiment Result

#### 4.2.1. Comparison of Convergence Precision of Different Algorithms

Table 2, Table 3, Table 4, Table 5, Table 6 and Table 7 show the optimization results of the Sphere function, the Schwefel function, the Rosenborck function, the Rastrigin function, the Ackley function and the Griewank function, respectively.

The tables above compare the convergence precision of the three algorithms with different functions, and the average value of 30 experiments is selected as the global optimal value of each algorithm. We can also know that D denotes the average value of different functions, and SD denotes standard deviation in the tables. Except the Schwefel function, the WGABC algorithm proposed in this paper is superior to the basic ABC algorithm and to the GABC algorithm in terms of convergence precision. The global minimum is in bold font, meanwhile, the convergence precision decreases as the dimension of D increases.

#### 4.2.2. Comparison of Convergence Speed of Different Algorithms

**Figure 2:**
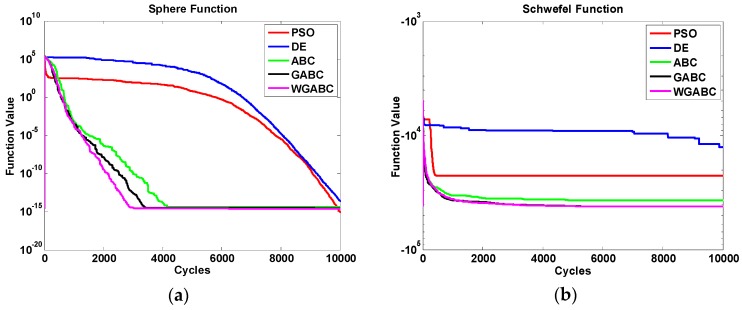

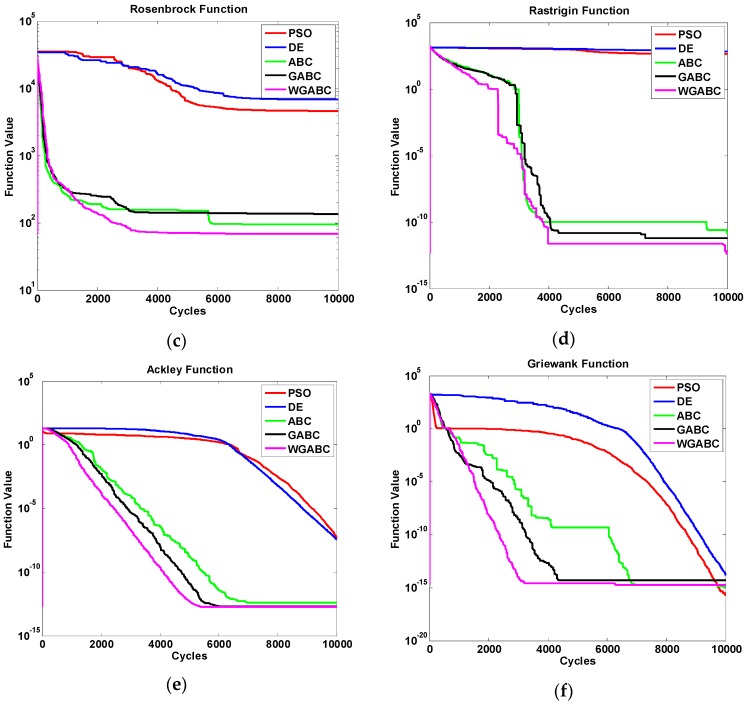
(**a**) The convergence speed diagram of Sphere function; (**b**) The convergence speed diagram of Schwefel function; (**c**) The convergence speed diagram of Rosenbrock function; (**d**) The convergence speed diagram of Rastrigin function; (**e**) The convergence speed diagram of Ackley function; (**f**) The convergence speed diagram of Griewank function.

In order to compare convergence speed of different algorithms, we carried on the simulation with the benchmark functions, wherein D is 100, and the number of food sources is 100. The number of particles is 100 in PSO algorithm, both of the coefficient of velocity updating formula is 2, and the dimension of the solution is 100. The number of populations is 100 in DE algorithm, the mutation rate is 0.5, crossover probability is 0.9, meanwhile, the dimension of the solution is 100. The number of iterations in all algorithms is 10,000. The convergence speeds of different algorithms are shown in Figure 2.

D is the dimension of the feasible solution, and in the experiments D = 100. In the convergence speed graph of the six functions above, the red curve represents the PSO algorithm, the blue curve represents the DE algorithm, the green curve represents the original ABC algorithm, the black curve represents the GABC algorithm literature [15] put forward, and the purple curve represents the WGABC algorithm we proposed. We can see from the diagram, in the process of obtaining the objective function value of six benchmark functions, WGABC as compared with other four kinds of algorithms has good convergence speed in the initial iteration and subsequent iterations, and also good robustness and convergence precision, so that it can improve the performance of ABC algorithm greatly.

## 5. Establishment of the Gas Transmission Model Considering Influence of Buildings

In order to prevent a harmful gas leak which can cause serious consequences, the chemical industrial park will install a gas sensor in accordance with the relevant standards, but the factors such as buildings still have some influence on the gas diffusion model, so the gas sensor deployed according to the conventional rules may not detect gas leaks. For the purpose of optimizing the deployment of gas sensors and improving the monitoring efficiency thereof, we first establish the Gaussian gas diffusion model considering the influence of building factors.

### 5.1. Gaussian Gas Diffusion Model

At present the gas diffusion model is mainly based on the Gaussian diffusion model, as shown below in Equation (6).

(6) $$c(x,y,z,H)=\frac{Q}{2\pi u\sigma_{y}\sigma_{z}}\exp(-\frac{y^{2}}{2\sigma_{y}{}^{2}})\left\{\exp[-\frac{{\left(z-H\right)}^{2}}{2\sigma_{y}{}^{2}}]+\exp[-\frac{{\left(z+H\right)}^{2}}{2\sigma_{z}{}^{2}}]\right\}$$

Wherein x is the axis of the diffusion direction that is consistent with the direction of the wind, y axis and x axis are perpendicular to each other in the horizontal plane, and z is perpendicular to the plane made by the y axis and x axis, Q denotes the leaking source intensity, the unit of which is kg/s, u means the wind speed, the unit of which is m/s, H is the height of the leakage source, $\sigma_{y}$ and $\sigma_{z}$ are the atmospheric diffusion parameters, or dilution factors, which are the functions of x, and the unit of which is m, the expressions are $\sigma_{Y}=c_{1}\times X^{p}$ and $\sigma_{Z}=c_{2}\times X^{g}$ respectively, so we can get the gas concentration at any point in diffusion space if we know the two parameters in the formulas above.

### 5.2. Fitting of Gas Diffusion Model Parameters in Combination with the Influence of Buildings

**Figure 3:**
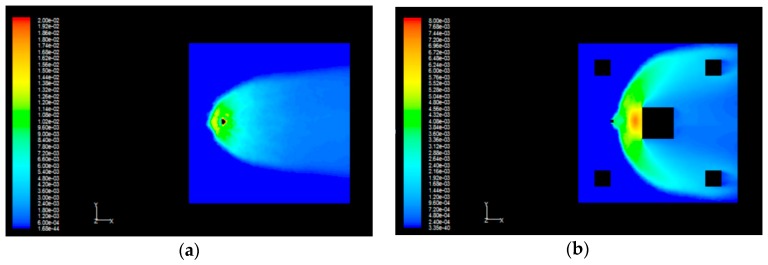
(**a**) The diagram of gas diffusion without the influence of buildings; (**b**) The diagram of gas diffusion with the influence of buildings.

In consideration of the differences in gas diffusion concentration caused by the influence of buildings, we use the theory of computational fluid dynamics (CFD) to simulate the diffused propane in a park. The simulation zone size is 50 m × 50 m × 10 m, the park zone includes five buildings which are numbered as building 1, building 2, building 3, building 4, and building 5, and the size of the building 1 is 10 m × 10 m × 5 m, the size of the rest four buildings is 5 m × 5 m × 5 m, and the size of leakage source is 1 m × 1 m × 2 m. We can get two gas diffusion models respectively with and without the influence of buildings. The leaking source has an intensity of 10.98 kg/s and a height of 2 m, namely H = 2 m. When the wind speed is 1.5 m/s, the gas diffusion conditions are shown in Figure 3.

As shown in Figure 3b, the left small square indicates a propane gas leakage source, the squares at four corners denote four buildings, and the big square is the building 1. We can see that the diffusion conditions are very different from each other irrespective of the influence of buildings, so the influence of the buildings on the gas diffusion model is of great significance to the gas sensor deployment in an actual park.

We set z = 0.6 m as the observation surface in the simulation, and select lots of points on the surface to get the corresponding concentration, as the measured gas concentration is the molar concentration having a unit of kmol/m^3^, the unit of the Gaussian diffusion concentration is g/m^3^, and the propane gas molecular weight is 44 g/mol, 1 kmol/m^3^ × 44 g/mol = 44,000 g/m^3^ according to the formula. For easy calculation, the unit of the gas diffusion concentration is set to kg/m^3^, so the molarity multiplied by the molecular mass is equal to the diffusion concentration. According to the values of these points, we can fit out the parameters $c_{1}$, $c_{2}$, p and g of the gas diffusion model based on WGABC algorithm.

The steps of the parameter fitting process are listed as follows:

1. Establishment of the objective function

For the estimation of gas diffusion model parameters, the most important thing is to establish the objective function. This paper uses the gas concentration difference between the prediction model and the measurement model as the objective function, which is minimized to obtain the optimal parameters.

The objective function is shown as follows:

(7) $$\min imize\;f_{1}(c_{1},p,c_{2},g)=\sum_{i=1}^{N}{\left(Y_{i}-\frac{Q}{2\pi u\sigma_{y}\sigma_{z}}\exp(-\frac{y^{2}}{2\sigma_{y}{}^{2}})\left\{\exp[-\frac{{\left(z-H\right)}^{2}}{2\sigma_{y}{}^{2}}]+\exp[-\frac{{\left(z+H\right)}^{2}}{2\sigma_{z}{}^{2}}]\right\}\right)}^{2}$$

where $Y_{i}$ is the concentration measured by simulation with the unit of kg/m^3^.

Using the prediction model, we can get the concentration predicted, and the unit is kg/m^3^, N denotes the density samples according to the different observation points, and $\sigma_{Y}=c_{1}\times X^{p}$, $\sigma_{Z}=c_{2}\times X^{g}$. Meanwhile, we use 57 density samples without the influence of buildings and 76 density samples with the influence of buildings, the algorithms of which are completed by MATLAB software.

2. Employed bee phase

In the Employed bee phase, the M food sources are randomly initialized to 4-dimensional matrixes which include initial parameters.

3. Cross and mutate the jth element of each initialized food source with the jth element of the neighbor food source randomly according to Equation (5).
4. Calculate the fitness of each food source according to the Equation (1) $[fitness_{1},fitness_{2}\cdots fitness_{M}]'$.
5. Calculate the probability of each food source according to the Equation (3).
6. Onlooker bee phase

Generate a number in the range of [0, 1] randomly, and compare the random number with the probability of each food source, if the probability of the random number is less than the food source, then cross and mutate the food source according to Equation (5).

7. Scout bee phase

If a food source still cannot converge after limit times of crossover and mutation, the food source would be abandoned, an initialized new food source is created and at the same time the scout bee becomes an employed bee.

Keep iterating until reaching the global optimal value and obtaining the corresponding coordinates.

The fitting parameters obtained using the WGABC algorithm are shown in Table 8 and Table 9. Both of them are the minimum values obtained by repeating the experiment for 30 times.

## 6. The Optimal Deployment of Gas Sensor Based on WGABC Algorithm

We can obtain the different gas diffusion model according to the parameters obtained in Section 5.2. The gas diffusion model without the influence of buildings is shown as follows:

(8) $$\begin{array}{l} C_{1}(x,y,z,H)=\frac{Q}{2\pi u*0.1209x^{67.2723}*1.3696x^{\left(-65.9386\right)}}\exp(-\frac{y^{2}}{2*{\left(0.1209x^{67.2723}\right)}^{2}})\\ \left\{\exp[-\frac{{\left(z-H\right)}^{2}}{2*{\left(0.1209x^{67.2723}\right)}^{2}}]+\exp[-\frac{{\left(z+H\right)}^{2}}{2*{\left(1.3696*x^{\left(-65.9386\right)}\right)}^{2}}]\right\} \end{array}$$

the gas diffusion model with the influence of buildings is shown as follows:

(9) $$\begin{array}{l} C_{2}(x,y,z,H)=\frac{Q}{2\pi u*2.4189x^{76.8196}*0.3187x^{\left(-75.9183\right)}}\exp(-\frac{y^{2}}{2*{\left(2.4189x^{76.8196}\right)}^{2}})\\ \left\{\exp[-\frac{{\left(z-H\right)}^{2}}{2*{\left(2.4189x^{76.8196}\right)}^{2}}]+\exp[-\frac{{\left(z+H\right)}^{2}}{2*{\left(0.3187x^{\left(-75.9183\right)}\right)}^{2}}]\right\} \end{array}$$

Make the Equation (8) as the prediction model and the Equation (9) as the measurement model, then we deploy the gas sensor optimally using WGABC algorithm, the specific steps are presented as follows:

1. Establish the experiment objective function

Based on the ABC optimization algorithm, the most important thing is to establish the objective function. This paper makes the average error of the prediction model and the measurement model as the objective function. There are N sensor nodes, the deployed locations of the nodes are used as the solution vector, and the average concentration error rate of the N sensors during the diffusion time is the objective function, which is made to be a minimum. The expression of the gas diffusion model without the influence of buildings is $C_{1}$, and the expression of the gas diffusion model with the influence of buildings is $C_{2}$.

The objective function is

(10) $$\min imize\;J=\frac{\sum_{1}^{N}\left(\frac{\left|C_{1}(x,y,z,H)-C_{2}(x,y,z,H)\right|}{C_{2}(x,y,z,H)}\right)}{N}$$

The objective function is expressed as the average concentration error rate of predicted values and measured values of each leakage source in the sensor locations, where N is the number of gas sensors. The parameters of the food sources are sensor location coordinates, the range of x and y is within [0, 50] m, the value of z is set to be 0.6 m.

2. Employed bee phase

In the employed bee phase, the M food sources are initialized randomly to D-dimensional matrixes, which include initial coordinates.

(11) $$P_{1}=[(x_{11},y_{11})\cdots (x_{1D},y_{1D})]$$

⋮

(12) $$P_{M}=[(x_{M1},y_{M1})\cdots (x_{MD},y_{MD})]$$

where

(13) $$\left(x_{1},y_{1})\cdots (x_{D},y_{D}\right)$$

⋮

(14) $$\left(x_{M1},y_{M1})\cdots (x_{MD},y_{MD}\right)$$

are the initial coordinates.

The remaining steps are the same as Section 5.2, so, we can refer to those contents.

## 7. Simulation Experiments and Analysis

### 7.1. Simulation Scenario and Parameter Settings

The experiment simulates a propane gas leakage in a chemical industrial park, wherein the simulation area is 50 m × 50 m, the mass release rate of the gas source is Q=10.98 kg/s, the wind speed is 1.5 m/s, and the wind is blown to the x positive axis, and the number of iterations is 2000 in every experiment. Because z = 0.6 m, the x and y coordinates should be enough.

### 7.2. The Influence of Different Algorithms on the Gas Diffusion Concentration Error Rate

**Figure 4:**
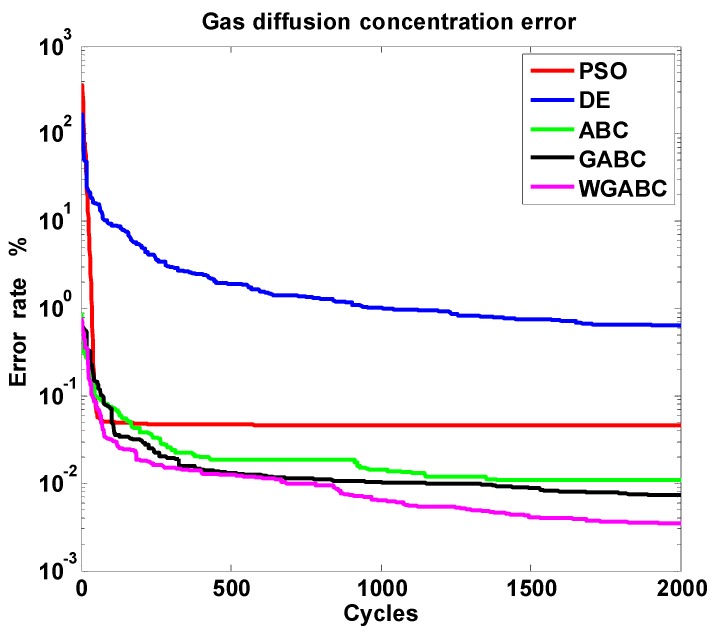
Diagram of error rate of the gas diffusion concentration.

We research the convergence speed of different algorithms on the error rate between the predict diffusion model and the measuring model, meanwhile, the number of sensor nodes is 20, which means the dimension of the solution is 40. The number of food sources is 20, the number of particles is 20 in PSO algorithm, the number of populations is 20 in DE algorithm. The wind speed is 1.5 m/s. According to the objective function Equation (10), the change trend of the gas diffusion error rate is shown in Figure 4.

As shown in Figure 4, the red curve represents the PSO algorithm, the blue curve represents the DE algorithm, the green curve represents the original ABC algorithm, the black curve represents the GABC algorithm literature [15] put forward, and the purple curve represents the WGABC algorithm we proposed. We can see the convergence speed and convergence precision of WGABC are better than the other four algorithms, and the experiment verifies that it is feasible to solve the problem of gas sensor optimum deployment using this improved algorithm.

### 7.3. Comparison of Monitored Concentration of Different Deployment Schemes

**Figure 5:**
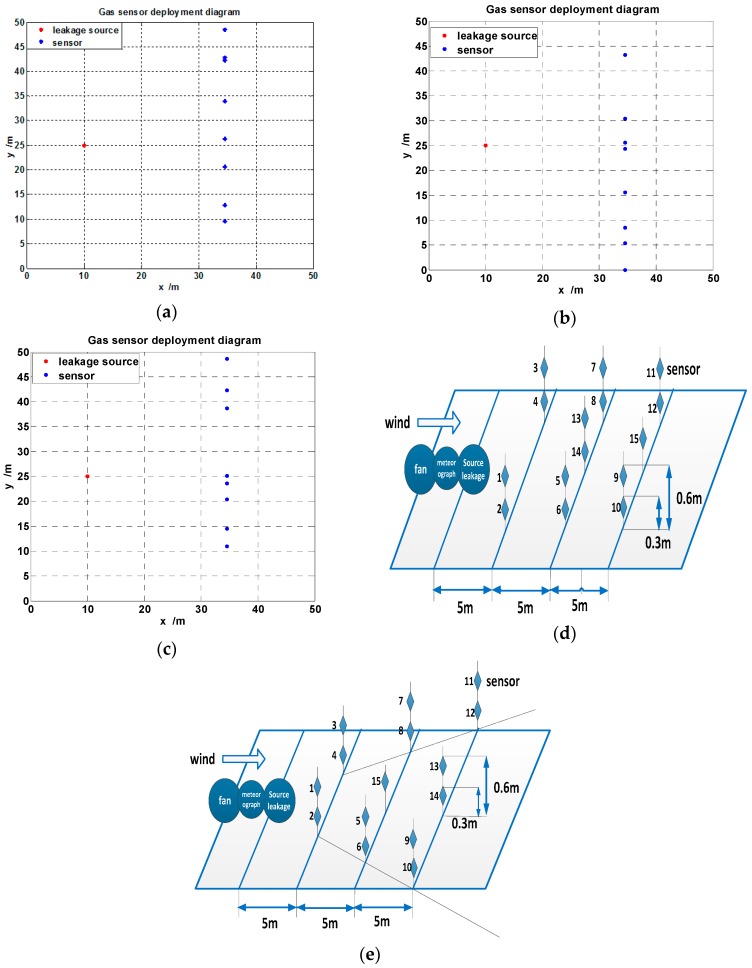
(**a**) The gas sensor deployment diagram based on WGABC algorithm; (**b**) The gas sensor deployment diagram based on PSO algorithm; (**c**) The gas sensor deployment diagram based on DE algorithm; (**d**) The rectangular gas sensor deployment diagram according to the standard; (**e**) The sector gas sensor deployment diagram according to the standard.

The optimal deployment scheme proposed in this paper is shown in Figure 5a, the deployment scheme based on PSO algorithm is shown in Figure 5b, the deployment scheme based on DE algorithm is shown in Figure 5c, and according to the Chinese GB50493-2009 and the Application Data Sheet ADS-001 Gas Sensor Placement Guidelines, multiple gas sensor monitoring networks are deployed usually in the form of the rectangle or sector, which are shown in Figure 5d,e.

As shown in Figure 5a, the coordinates of eight sensors in the optimal deployment scheme are shown in Table 10.

In consideration of the influence of buildings on the gas diffusion, the influence of buildings can be prevented if the gas sensors are deployed at a horizontal distance of 35 m, so the method we proposed is very reasonable.

As shown in Figure 5b, the coordinates of eight sensors in the optimal deployment scheme are shown in Table 11.

As shown in Figure 5c, the coordinates of eight sensors in the optimal deployment scheme are shown in Table 12.

We divided the simulation area into some grids with size of 5 m × 5 m, and get the gas concentration of every grid point using the theory of CFD with simulation.

The measured concentration is obtained from the grid point which is closest to the position of sensors, and the measured concentration of the deployed sensor based on WGABC algorithm is shown in Table 13.

The measured concentration of the deployed sensor based on PSO algorithm is shown in Table 14.

The measured concentration of the deployed sensor based on PSO algorithm is shown in Table 15.

According to Figure 5d, the space between each row and each column is 5 m, but due to the influence of the buildings, we need to take the buildings into account, so the coordinates of the sensors are shown in Table 16.

According to Figure 5e, the angle between the edge of the sector and horizontal direction is 30 degrees. The average space of the sector edge is 5 m, where the sensors are deployed. However, due to the influence of the buildings, we need to take the buildings into account, and the coordinates of the sensors are shown in Table 17.

### 7.4. The Alarm Concentration and Optimization Standards

According to the Chinese GB50493-2009, the lower explosive limit (LEL) of propane gas is 2.1% (volume ratio mixed with air), the upper explosive limit (ULE) of propane gas is 9.5%. Therefore, the optimization criterion of this paper is that the more sensors that monitor the alarm concentration, the better the deployment scheme.

### 7.5. The Performance Analysis of Different Deployment Schemes

We can know whether the detected concentration reaches the alarm concentration or not according to the formula V=C×22.4L/mol , so the gas volume fractions of five schemes are shown in Table 18, Table 19, Table 20, Table 21 and Table 22, respectively.

**Figure 6:**
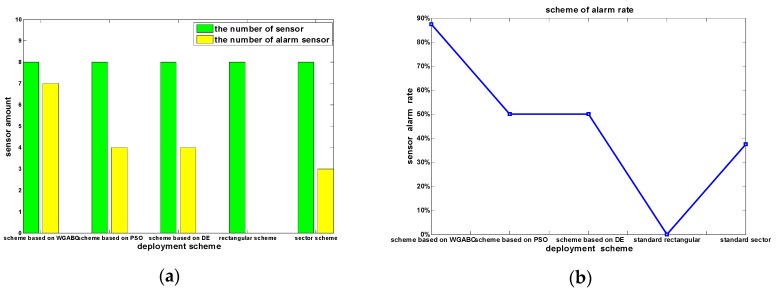
(**a**) The number of alarm sensors in different deployment schemes; (**b**) The alarm rate curves in different deployment schemes.

The concentration volume fractions that reached the alarm concentration fraction are in bold font in the tables above. So we can know that the number of alarm sensors is seven in the scheme we proposed, and the alarm rate is 87.5%. The number of alarm sensors is four in the scheme based on PSO algorithm, and it is the same as the scheme based on DE algorithm, and both of the alarm rate in the two schemes is 50%. The number of alarm sensors is 0 in the rectangular scheme according to the standard, the number of alarm sensors is three in the sector scheme according to the standard, and the alarm rate is only 37.5%. The performance difference of different schemes is shown in Figure 6, from which we can know directly that the optimization deployment scheme this paper proposed can improve the efficiency of monitoring significantly as compared with the other deployment schemes. So this deployment method is feasible and practical to some extent.

## 8. Conclusions

In the chemical industrial park, the gas sensor installed in accordance with the traditional standards may not detect gas leakage efficiently due to the influence of buildings, so it is very necessary to adopt the gas sensor network deployment method designed in consideration of both the gas characteristic and environment characteristic, meanwhile, the method can provide inspiration and guidance for current layout schemes. In this paper, we first propose the improved ABC algorithm called Weighted Global ABC algorithm considering global factors and aimed at solving the problem of slow convergence speed in the search stage of the solution search equation without considering global factors. We not only consider the global factors but also give an expression of the global weight in front of the global factors, which provide the algorithm with good global optimization characteristic at the beginning of the iteration and good local optimization characteristic at the end of the iteration. Then we verified the effectiveness of the algorithm on different benchmark functions compared with PSO algorithm, DE algorithm, ABC algorithm, and GABC algorithm. Then we get the gas diffusion concentration using the theory of CFD by way of simulation, and fitted out the parameters of the gas diffusion with the influence of buildings and without the influence of buildings based on WGABC algorithm, and obtained two gas diffusion models. Finally, we proposed a new type of deployment optimization scheme using WGABC algorithm, and the experiment shows that the monitoring efficiency of the proposed scheme is better than the other schemes, the monitoring efficiency of the proposed scheme is 87.5%, meanwhile, those of the others are 50%, 0, and 37.5% respectively, so the improvement is obvious. Now this thesis mainly takes into consideration the impact of buildings on the gas diffusion concentration, and the future studies may be focused on the gas sensor deployment optimization under the circumstances that topography has an influence on gas diffusion.

## Figures and Tables

**Table 1:** Expression of six test functions.

Function	Range	Formulate
Sphere	[−100, 100]^D^	$f(x)=\sum_{i=1}^{D}x_{i}{}^{2}$
Schwefel	[−500, 500]^D^	$f(X)=418.9829\times D-\sum_{i=1}^{E}X_{i}\sin(\sqrt{\left|X_{i}\right|})$
Rosenbrock	[−2.048, 2.048]^D^	$f(x)=\sum_{i=1}^{D-1}\left[100{\left(x_{i+1}-x_{i}{}^{2}\right)}^{2}+{\left(x_{i}-1\right)}^{2}\right]$
Rastrigin	[−5.12, 5.12]^D^	$f(x)=\sum_{i=1}^{D}\left[x_{i}{}^{2}-10\cos(2\pi x_{i})+10\right]$
Ackley	[−32.768, 32.768]^D^	$f(x)=-20\exp(-0.2\sqrt{\frac{1}{n}\sum_{i=1}^{D}x_{i}{}^{2}})-\exp(\frac{1}{n}\sum_{i=1}^{D}\cos(2\pi x_{i}))+20+e$
Griewank	[−600, 600]^D^	$f(x)=\frac{1}{4000}(\sum_{i=1}^{D}{\left(x_{i}-100\right)}^{2})-(\prod_{i=1}^{D}\cos(\frac{x_{i}-100}{\sqrt{i}}))+1]$

**Table 2:** The mean and standard deviation of the Sphere function on the different algorithms.

Algorithm	Sphere Function			
	D = 30		D = 60	
	Mean	SD	Mean	SD
PSO	5.999 × 10^−14^	4.8556 × 10^−14^	3.5180 × 10^−4^	2.7372 × 10^−4^
DE	2.2675 × 10^−6^	2.2000 × 10^−6^	19.5393	18.9164
ABC	9.4601 × 10^−16^	2.1597 × 10^−16^	4.5907 × 10^−13^	2.4636 × 10^−13^
GABC (C = 1.5)	7.4670 × 10^−16^	7.238 × 10^−17^	1.8251 × 10^−15^	2.766 × 10^−16^
WGABC	**6.1524 × 10^−16^**	**6.513 × 10^−17^**	**1.6005 × 10^−15^**	**2.399 × 10^−16^**

**Table 3:** The mean and standard deviation of the Schwefel function on the different algorithms.

Algorithm	Schwefel Function			
	D = 30		D = 60	
	Mean	SD	Mean	SD
PSO	−5.0071 × 10^3^	1.4658 × 10^3^	−1.2760 × 10^4^	1.0255 × 10^3^
DE	−1.0987 × 10^4^	513.3913	−1.6961 × 10^4^	1.7213 × 10^3^
ABC	−1.2482 × 10^3^	6.282 × 10^2^	**−2.4158 × 10^4^**	5.403 × 10^4^
GABC (C = 1.5)	−1.8295 × 10^3^	**8.4245**	−3.7122 × 10^3^	**2.8 × 10**
WGABC	**−1.8923 × 10^6^**	5.6712 × 10^3^	−3.556 × 10^2^	1.0685 × 10^3^

**Table 4:** The mean and standard deviation of the Rosenbrock function on the different algorithms.

Algorithm	Rosenbrock Function			
	D = 2		D = 3	
	Mean	SD	Mean	SD
PSO	1.44 × 10^−2^	8.7 × 10^−3^	0.1141	0.0817
DE	0.0020	0.0017	0.0248	0.0302
ABC	1.32 × 10^−2^	1.60 × 10^−2^	3.87 × 10^−2^	4.92 × 10^−2^
GABC (C = 1.5)	3.1417 × 10^−4^	2.8765 × 10^−4^	7.6 × 10^−3^	7.7 × 10^−3^
WGABC	**4.3581 × 10^−6^**	**3.2239 × 10^−6^**	**3.8486 × 10^−5^**	**3.3463 × 10^−5^**

**Table 5:** The mean and standard deviation of the Rastrigin function on the different algorithms.

Algorithm	Rastrigin Function			
	D = 30		D = 60	
	Mean	SD	Mean	SD
PSO	65.3188	18.5433	302.8413	60.5854
DE	37.2176	10.4854	117.1389	18.0191
ABC	4.5474 × 10^−13^	**1.1182 × 10^−13^**	1.4877	0.9272
GABC (C = 1.5)	4.3752 × 10^−13^	5.1613 × 10^−13^	2.5258	1.3028
WGABC	**3.2990 × 10^−13^**	2.6611 × 10^−13^	**9.9893 × 10^−9^**	**1.7378 × 10^−8^**

**Table 6:** The mean and standard deviation of the Ackley function on the different algorithms.

Algorithm	Ackley Function			
	D = 30		D = 60	
	Mean	SD	Mean	SD
PSO	1.4879 × 10^−7^	7.9102 × 10^−8^	0.1019	0.2347
DE	1.3686	4.5629 × 10^−4^	4.3042	1.5498
ABC	6.6969 × 10^−14^	9.6903 × 10^−15^	3.0244 × 10^−6^	2.0320 × 10^−6^
GABC (C = 1.5)	4.9560 × 10^−14^	6.1638 × 10^−15^	1.4945 × 10^−9^	5.9658 × 10^−10^
WGABC	**4.6008 × 10^−14^**	**7.7917 × 10^−15^**	**1.0126 × 10^−10^**	**7.0351 × 10^−11^**

**Table 7:** The mean and standard deviation of the Griewank function on the different algorithms.

Algorithm	Griewank Function			
	D = 30		D = 60	
	Mean	SD	Mean	SD
PSO	9.8921 × 10^−15^	1.9479 × 10^−14^	4.8049 × 10^−6^	3.1064 × 10^−6^
DE	1.6315 × 10^−5^	1.0805 × 10^−5^	1.0115	0.2862
ABC	5.1891 × 10^−15^	3.9323 × 10^−15^	2.7377 × 10^−13^	1.1152 × 10^−13^
GABC (C = 1.5)	6.3148 × 10^−16^	5.3762 × 10^−16^	5.3657 × 10^−15^	3.5802 × 10^−15^
WGABC	**9.9920 × 10^−17^**	**1.0724 × 10^−16^**	**5.4137 × 10^−16^**	**3.2162 × 10^−16^**

**Table 8:** The parameters of the gas diffusion model without the influence of buildings.

No Buildings Exist			
$c_{1}$	p	$c_{2}$	g
0.1209	67.2723	1.3696	−65.9386

**Table 9:** The parameters of the gas diffusion model with the influence of buildings.

Buildings Exist			
$c_{1}$	p	$c_{2}$	g
2.4189	76.8196	0.3187	−75.9183

**Table 10:** The coordinates of gas sensor in optimal deployment scheme based on WGAC algorithm.

	S1	S2	S3	S4	S5	S6	S7	S8
X (m)	34.54	34.54	34.54	34.54	34.54	34.54	34.54	34.54
Y (m)	42.76	33.90	9.56	46.44	26.34	42.22	20.65	12.81
Z (m)	0.60	0.60	0.60	0.60	0.60	0.60	0.60	0.60

**Table 11:** The coordinates of gas sensor in optimal deployment scheme based on PSO algorithm.

	A1	A2	A3	A4	A5	A6	A7	A8
X (m)	34.54	34.54	34.54	34.54	34.54	34.54	34.54	34.54
Y (m)	30.39	8.50	0	43.25	24.35	15.56	25.58	5.38
Z (m)	0.60	0.60	0.60	0.60	0.60	0.60	0.60	0.60

**Table 12:** The coordinates of gas sensor in optimal deployment scheme based on DE algorithm.

	B1	B2	B3	B4	B5	B6	B7	B8
X (m)	34.54	34.54	34.54	34.54	34.54	34.54	34.54	34.54
Y (m)	14.48	23.56	25.07	10.91	38.70	20.34	48.65	42.35
Z (m)	0.60	0.60	0.60	0.60	0.60	0.60	0.60	0.60

**Table 13:** The measured concentration of deployment scheme based on WGABC algorithm.

**Sensor**	**S1**	**S2**	**S3**	**S4**
C (kmol/m^3^)	1.61 × 10^−3^	1.07 × 10^−3^	1.50 × 10^−3^	2.01 × 10^−3^
**Sensor**	**S5**	**S6**	**S7**	**S8**
C (kmol/m^3^)	6.75 × 10^−4^	1.61 × 10^−3^	1.61 × 10^−3^	1.50 × 10^−3^

**Table 14:** The measured concentration of deployment scheme based on PSO algorithm.

**Sensor**	**A1**	**A2**	**A3**	**A4**
C (kmol/m^3^)	7.11 × 10^−4^	1.50 × 10^−3^	7.44 × 10^−7^	2.01 × 10^−3^
**Sensor**	**A5**	**A6**	**A7**	**A8**
C (kmol/m^3^)	6.75 × 10^−4^	9.62 × 10^−4^	6.75 × 10^−4^	1.92 × 10^−3^

**Table 15:** The measured concentration of deployment scheme based on DE algorithm.

**Sensor**	**B1**	**B2**	**B3**	**B4**
C (kmol/m^3^)	9.62 × 10^−4^	6.85 × 10^−4^	6.74 × 10^−4^	1.50 × 10^−3^
**Sensor**	**B5**	**B6**	**B7**	**B8**
C (kmol/m^3^)	1.61 × 10^−3^	5.68 × 10^−4^	2.62 × 10^−5^	2.01 × 10^−3^

**Table 16:** The coordinates of the sensors and the corresponding concentration according to the standard rectangular deployment method.

	R1	R 2	R 3	R 4	R 5	R 6	R 7	R 8
X (m)	15	15	35	35	35	40	40	40
Y (m)	20	30	20	25	30	20	25	30
Z (m)	0.60	0.60	0.60	0.60	0.60	0.60	0.60	0.60
C (kmol/m^3^)	4.69 × 10^−3^	4.62 × 10^−3^	5.68 × 10^−4^	6.75 × 10^−4^	7.11 × 10^−4^	5.11 × 10^−4^	6.08 × 10^−4^	5.844 × 10^−4^

**Table 17:** The coordinates of the sensors and the corresponding concentration according to the standard sector deployment method.

	D1	D 2	D 3	D 4	D 5	D 6	D 7	D 8
X (m)	15	15	20	20	25	25	30	30
Y (m)	22.11	27.88	19.23	30.77	16.34	33.66	20	30
Z (m)	0.60	0.60	0.60	0.60	0.60	0.60	0.60	0.60
C (kmol/m3)	4.69 × 10^−3^	4.63 × 10^−3^	3.71 × 10^−3^	4.31 × 10^−3^	1.74 × 10^−3^	2.19 × 10^−3^	5.51 × 10^−4^	7.24 × 10^−4^

**Table 18:** The measured concentration volume fraction of deployment scheme based on WGABC algorithm.

Sensor	S1	S2	S3	S4	S5	S6	S7	S8
V (%)	**3.61%**	**2.40%**	**3.36%**	**4.50%**	1.51%	**3.61%**	**3.61%**	**2.40%**

**Table 19:** The measured concentration volume fraction of deployment scheme based on PSO algorithm.

Sensor	A1	A2	A3	A4	A5	A6	A7	A8
V (%)	1.59%	**3.36%**	0.0017%	**4.50%**	1.51%	**2.15%**	1.51%	**4.30%**

**Table 20:** The measured concentration volume fraction of deployment scheme based on DE algorithm.

Sensor	B1	B2	B3	B4	B5	B6	B7	B8
V (%)	**2.15%**	1.51%	1.51%	**3.36%**	**3.60%**	1.27%	0.058%	**4.50%**

**Table 21:** The measured concentration volume fraction of the rectangular deployment scheme.

Sensor	R1	R 2	R 3	R 4	R 5	R 6	R 7	R 8
V (%)	10.51%	10.35%	1.27%	1.51%	1.59%	1.14%	1.36%	1.31%

**Table 22:** The measured concentration volume fraction of the sector deployment scheme.

Sensor	D1	D 2	D 3	D 4	D 5	D 6	D 7	D 8
V (%)	10.51%	10.37%	**8.31%**	9.65%	**3.83%**	**4.91%**	1.23%	1.62%
